# Loneliness and social isolation during the COVID-19 pandemic

**DOI:** 10.1017/S1041610220000988

**Published:** 2020-05-26

**Authors:** Tzung-Jeng Hwang, Kiran Rabheru, Carmelle Peisah, William Reichman, Manabu Ikeda

**Affiliations:** 1 Department of Psychiatry, College of Medicine and National Taiwan University Hospital, National Taiwan University, Taipei, Taiwan; 2 Department of Psychiatry, Faculty of Medicine, University of Ottawa, Ottawa, Canada; 3 School of Psychiatry, University New South Wales; and Discipline Psychiatry, Sydney University, Sydney, Australia; 4 Department of Psychiatry, Faculty of Medicine, University of Toronto, Toronto, Canada; 5 Department of Psychiatry, Osaka University Graduate School of Medicine, Osaka, Japan

## Introduction

The COVID-19 pandemic has led to implementation of unprecedented “social distancing” strategies crucial to limiting the spread of the virus. In addition to quarantine and isolation procedures for those who have been exposed to or infected with COVID-19, social distancing has been enforced amongst the general population to reduce the transmission of COVID-19.

The risk of COVID-19 infection is greater for older adults over the age of 60 years who are at a heightened risk of severe illness, hospitalization, intensive care unit admission, and death (US CDC, 2020). According to the Centre for Evidence-Based Medicine, the case fatality rate (CFR) is about 4% for patients over 60 years old, 8% for patients over age 70 years, and approximately 15% for patients over the age of 80 (Oxford COVID-19 Evidence Service, 2020). This compares with CFR of 0.0026%–0.3% in those under age 45.

However, there is a high cost associated with the essential quarantine and social distancing interventions for COVID-19, especially in older adults, who have experienced an acute, severe sense of social isolation and loneliness with potentially serious mental and physical health consequences. The impact may be disproportionately amplified in those with pre-existing mental illness, who are often suffering from loneliness and social isolation prior to the enhanced distancing from others imposed by the COVID-19 pandemic public health measures.

Older adults are also more vulnerable to social isolation and loneliness as they are functionally very dependent on family members or supports by community services. While robust social restrictions are necessary to prevent spread of COVID-19, it is of critical importance to bear in mind that social distancing should not equate to social disconnection.

The present position paper aims to describe the nature of loneliness and social isolation among older persons, its effect on their health, and ways to cope with loneliness and social isolation during the COVID-19 pandemic.

## Loneliness and social isolation

Loneliness and social isolation frequently co-occur and are all too common in older adults. While the term loneliness refers to subjective feelings, social isolation is defined by the level and frequency of one’s social interactions. As a generally accepted concept, loneliness is defined as the subjective feeling of being alone, while social isolation describes an objective state of individuals’ social environments and interactional patterns. Studies suggest that while loneliness and social isolation are not equal to each other, both can exert a detrimental effect on health through shared and different pathways.

Prior to the COVID-19 pandemic, loneliness and social isolation were so prevalent across Europe, the USA, and China (10–40%) (Leigh-Hunt *et al.*, 2017; Xia and Li, 2018) that it was described as a “behavioral epidemic” (Jeste *et al.*, 2020). The situation has only worsened with the restrictions imposed to contain viral spread.

## Physical and mental health impacts

Loneliness is associated with various physical and mental repercussions, including elevated systolic blood pressure and increased risk for heart disease. Both loneliness and social isolation have been associated with an increased risk for coronary artery disease-associated death, even in middle-aged adults without a prior history of myocardial infarction (Heffner *et al.*, 2011; Steptoe *et al.*, 2013). Furthermore, research has shown that both loneliness and social isolation are independent risk factors for higher all-cause mortality (Yu *et al.*, 2020).

Being lonely has several adverse impacts on mental health. Reduced time in bed spent asleep (7% reduced sleep efficiency) and increased wake time after sleep onset have been related to loneliness (Cacioppo *et al.*, 2002; Fässberg *et al.*, 2012). Increased depressive symptomatology may also be caused by loneliness, along with poor self-rated health, impaired functional status, vision deficits, and a perceived negative change in the quality of one’s life (Lee *et al.*, 2019). A systematic review of suicide risk also found that loneliness is associated with both suicide attempts and completed suicide among older adults (Fässberg *et al.*, 2012). Loneliness, along with depressive symptoms, are related to worsening cognition over time. A systematic review concluded that loneliness and social isolation were significantly associated with incident dementia (Kuiper *et al.*, 2015).

The proposed mechanism for the adverse health impacts of loneliness focuses on the physiological stress response (such as increased cortisol) (Xia and Li, 2018). Abnormal stress responses lead to adverse health outcomes. For social isolation, the mechanism may be related to behavioral changes, including an unhealthy lifestyle (such as smoking, alcohol consumption, lower physical activity, poor dietary choices, and noncompliance with medical prescription) (Kobayashi and Steptoe, 2018; Leigh-Hunt *et al.*, 2017). A smaller social network with less medical support exacerbates these conditions. Recognizing and developing a better understanding of these possible mechanisms should help us to design the most impactful interventions.

## Tips for preventing the detrimental effect of loneliness and social isolation

There are established ways to maintain feelings of being connected to others despite having to maintain social distancing. By organizing our activities every single day, we can become more resistant to the onset of feelings of loneliness. For older adults, some tips are as follows.

### Keep connections

- *Spend more time with your family*. Utilize opportunities offered by the pandemic. Before the pandemic, some family members may have been distracted by work and school commitments, but now they may have more time at home and a higher degree of freedom to connect with older loved ones. In the era of social distancing, quality interactions using physical distancing of at least two meters along with the use of personal protective equipment such as masks enable contact with family members. This is vitally helpful to defend against loneliness.
- *Maintain social connections with technology*. Along with the telephone, technology has changed the way people interact with each other. Social media platforms such as Facebook, Skype, Twitter, LINE, and Instagram enable people to stay connected in a variety of ways. Many older adults, however, may not be as familiar with these new technologies, and this style of interaction may not effectively serve their emotional needs. We can help older family members and friends to overcome such technology barriers. Online video chat is easier to use and sufficiently conveys nonverbal cues so that people can feel more engaged. Even without new technology available, communication through phone services is beneficial too. Conversations with a regular schedule through online or phone services with family members and loved ones can be helpful for older adults.

### Maintain basic needs and healthy activities

- *Ensure basic needs are met*. Family and carers should ensure food, medication, and mask accessibility for older adults, especially those who live alone.
- *Structure every single day*. To stay confined at home for much of every day is a psychological challenge for many people. When most outdoor activities are not available, it is not easy to maintain a regular daily schedule. However, we can encourage and support engagement with activities deemed pleasurable by the older person with benefits for physical, mental, and spiritual well-being. Regular scheduling is especially supportive for older people at risk of delirium, which is characterized by a disturbance of circadian rhythm. Television and YouTube channels adapted for older adults with proper physical and mental programs (e.g. exercise programs, mindfulness practice, and music programs) can also be very useful.
- *Maintain physical and mental activities*. Exercise has benefits for physical and psychological health (specifically for mood and cognition). There is evidence that regular engagement in mentally challenging and new activities may reduce the risk of dementia. Although we may not be able to exercise together as before, we should maintain physical activities at the individual level. Besides, these personal physical activities can be performed at a group level by setting a common goal, sharing our progress, or creating a friendly competition via social media.
- *Pursue outdoor activities while following the guidance of social distancing*. Brief outdoor activities are usually still possible and beneficial to health. One can feel much better as a result of sunlight exposure and the ability to see other people while still maintaining physical distancing.

### Manage emotions and psychiatric symptoms

- *Manage cognition, emotion, and mood*. Loneliness is often associated with negative thoughts (cognitions). Moreover, anxiety and depression may cause social withdrawal which will exacerbate the loneliness and isolation associated with social distancing. Acquiring reliable information about the pandemic helps avoid unnecessary worry and negative rumination. Conscious breathing, meditation, and other relaxation techniques are helpful for the mind and body and can decrease one’s level of anxiety and depression. Emotional support for family members and friends is especially important during this harsh pandemic period, but one should not hesitate to seek help as well.
- *Pay attention to psychiatric symptoms*. The pandemic is quite stressful for every individual, and the significant stress can precipitate the occurrence or recurrence of mental disorders in some people, especially vulnerable older people. Depression, anxiety, and sleep disturbance are common, especially when one is under quarantine or self-isolation. Other symptoms include anger, irritability, and compulsive behaviors, such as repeated washing and cleaning. Furthermore, the experiences of social isolation and quarantine may bring back post-traumatic stress disorder symptoms for those previously exposed to other related events such as the severe acute respiratory syndrome and Middle East respiratory syndrome epidemics (Hawryluck *et al.*, 2004). Online screening tools and rating scales can help us to understand the magnitude of the impact on our mental health. People with existing psychiatric disorders and their family members should pay special attention to their mental health and follow important tips to prevent worsening of symptoms. Medical assistance should always be sought when necessary, particularly in response to the expression of suicidal ideation. Those taking prescribed psychiatric medications should make sure that their supply is adequate, despite the limitations imposed by social distancing and the difficulty in visiting the pharmacy. Government agencies, social service organizations, and healthcare providers should consider offering online psychological services (or at least phone services) to those psychogeriatric patients who need medical advice during the social isolation period.
- *Take special care of older people with dementia and their family carers*. The world and the way people live have significantly been disrupted in response to the COVID-19 pandemic. Changes are always stressful and require people to adapt. However, people with dementia have compromised adaptive function, and the pandemic may aggravate negative emotions and invoke behavioral and psychological symptoms. Recognizing that people with dementia may find it difficult to understand and comply with social distancing, caregivers should try to give instructions on hand hygiene, social distancing, and other protective measures in a simple, straightforward, and understandable way. Regular daily schedules and activities should be arranged and individually tailored to the dementia patient’s interests. Family carers might be under especially severe levels of stress and feel even more isolated and alone. More detailed information on the unique aspects of the pandemic’s effects on dementia caregiving is available on the Alzheimer’s Disease International website (Alzheimer’s Disease International, 2020).

## Conclusion

The societal impact of the COVID-19 pandemic has been broad and very challenging. No aspect of normal societal functioning has been spared. Quarantine and social distancing are necessary measures to prevent the virus from spreading but also lead to elevated levels of loneliness and social isolation, which in turn produce physical- and mental-health related repercussions. Adopting appropriate steps to keep social and familial connections, maintain healthy activities, and manage emotions and psychiatric symptoms can help relieve the adverse consequences of loneliness and isolation. The pandemic has illuminated the pre-existing threat to well-being that older adults frequently experience with social isolation and loneliness. Perhaps we can use this moment to commit ourselves to addressing these unfortunate aspects of life for older adults in the post-pandemic period, for example, developing virtual health care, new technology, and government policy.

On the May 23, 2020, in collaboration with INTERDEM, IPA ran a webinar program addressing this very issue: “COVID-19, social distancing and its impact on social and mental health of the elderly population.”
